# Three-dimensional growth sensitizes breast cancer cells to treatment with ferroptosis-promoting drugs

**DOI:** 10.1038/s41419-023-06106-2

**Published:** 2023-09-01

**Authors:** Sandhya Chipurupalli, Peijia Jiang, Xiaoyang Liu, Julia Linhares Santos, Paola Marcato, Kirill V. Rosen

**Affiliations:** 1 Departments of Pediatrics & Biochemistry and Molecular Biology, Dalhousie University, Halifax, NS Canada; 2 Department of Pathology, Dalhousie University, Halifax, NS Canada

**Keywords:** Breast cancer, Cell death

## Abstract

Drugs causing ferroptosis, iron-mediated cell death, represent promising tools for cancer treatment. While exploring the effect of these drugs on breast cancer (BC), we found that a ferroptosis-inducing drug erastin dramatically inhibits tumorigenicity of human BC cells in mice but when used at a concentration known to effectively kill other cell types only modestly reduces such growth in 2D monolayer culture. BCs grow in vivo as 3D masses, and we found that ferroptosis inducers erastin and sulfasalazine inhibit growth of multiple human BC cell lines in 3D culture significantly stronger than in 2D culture. To understand the mechanism of this differential effect, we found that ferroptosis inducers upregulate mRNAs encoding multiple direct and indirect autophagy stimulators, such as ATG16L2, ATG9A, ATG4D, GABARAP, SQSTM/p62, SEC23A and BAX, in tumor cells growing in 2D but not in 3D culture. Furthermore, these drugs promoted autophagy of tumor cells growing in a 2D but not in a 3D manner. We observed that pharmacological inhibition of autophagy-stimulating protein kinase ULK1 or RNA interference-mediated knockdown of autophagy mediator ATG12 significantly sensitized tumor cells to erastin treatment in 2D culture. We also found that ferroptosis-promoting treatments upregulate heme oxygenase-1 (HO-1) in BC cells. HO-1 increases cellular free iron pool and can potentially promote ferroptosis. Indeed, we observed that HO-1 knockdown by RNA interference reversed the effect of ferroptosis inducers on BC cell 3D growth. Hence, the effect of these drugs on such growth is mediated by HO-1. In summary, autophagy triggered by ferroptosis-promoting drugs reduces their ability to kill BC growing in a 2D manner. This protection mechanism is inhibited in BC cells growing as a 3D mass, and ferroptosis-promoting drugs kill such cells more effectively. Moreover, this death is mediated by HO-1. Thus, ferroptosis induction represents a promising strategy for blocking 3D BC growth.

## Introduction

Breast cancers (BCs) often develop resistance to therapies and patients invariably succumb to the resulting metastatic disease [1]. Hence, novel BC therapies are needed. Unlike normal breast epithelial cells that grow in vivo in a 2D manner, attached to the extracellular matrix (ECM), BCs typically grow as 3D multicellular masses [2]. Moreover, detachment from the ECM kills non-malignant breast epithelial cells, whereas 3D BC cell masses are composed of viable cells, and such viability is required for BC progression [3]. Therefore, the ability of BC cells to grow in a 3D manner represents a critical target for BC therapies.

Ferroptosis is iron-catalyzed necrosis driven by polyunsaturated fatty acid peroxidation and the formation of lipid reactive oxygen species (ROS) in mammalian cell membranes [4]. This peroxidation destroys the membranes and causes cell death [4]. Glutathione peroxidase 4 (GPX4) is a major ferroptosis inhibitor, and a cysteine-containing tripeptide glutathione is a critical GPX4 cofactor. Glutathione production is mediated by the system X_C_^−^ that imports cystine, the oxidized cysteine form, to the cell, where cystine is converted to cysteine required for glutathione synthesis [4]. System X_C_^−^ consists of proteins SLC7A11 and SLC3A2. Various drugs cause ferroptosis by inhibiting this system. For example, a drug erastin inactivates SLC3A2 and blocks cysteine import [4]. This inhibits GPX4, and cellular phospholipid hydroperoxides are converted to phospholipid radicals in Fe^2+^-dependent manner which ultimately destroys the cell membrane [4].

Tumor cells typically have higher ROS levels than normal cells and ROS promote cancer [5]. Even though ROS levels are high in tumor cells, activation of various antioxidant systems prevents these levels from exceeding a certain threshold [5]. If the threshold is exceeded, e.g., in response to ferroptosis-inducing drugs, the cells die [5]. Moreover, tumor cells often have higher iron levels than normal cells which makes them hypersensitive to ferroptosis-inducing drugs [4]. Therefore, the use of such agents could represent an effective strategy for preferential tumor cell killing [5]. Of note, clinically approved drugs, including sulfasalazine, artesunate, lovastatin and dihydroartemisinin cause ferroptosis [6]. It was proposed that due to this ability, such agents could be repurposed for cancer treatment [6].

Autophagy is a process of degradation of the cellular content mediated by the vacuoles termed autophagosomes [7]. Autophagy allows cells to adapt to various stress types, e.g., by providing elements for biosynthesis of essential macromolecules or eliminating damaged macromolecules or organelles [7]. Autophagy can inhibit or stimulate ferroptosis. For example, autophagy can protect lung cancer, glioblastoma or hepatocellular carcinoma cells as well as neurons from ferroptosis [8–11] but promotes ferroptosis of other cell types, e.g., fibroblasts [12]. Factors determining whether autophagy stimulates or blocks ferroptosis are not well understood.

We show here that ferroptosis-promoting agents robustly promote autophagy of BC cells growing in a 2D manner and that autophagy protects them from ferroptosis. In contrast, these drugs induce autophagy much less efficiently in the cells growing in a 3D manner and kill them more potently than those growing in a 2D manner. Thus, induction of ferroptosis represents a promising strategy for blocking 3D BC growth.

## Materials and methods

### Materials

SBI-0206965 was from Cell Signalling Technology (Danvers, MA, USA), bafilomycin A1 and sulfasalazine, from Sigma-Aldrich (St. Louis, MO, USA), Matrigel, from VWR (Mississauga, ON, Canada) and erastin, from Tocris Bioscience (Toronto, ON, Canada). Primers for quantitative (q)PCR of mRNAs encoding autophagy mediators were from RealTimePrimers (Elkins Park PA, USA)

### Cell culture

BT-474, AU-565, MDA-MB486 and HCC-1806 were from (American Type Culture Collection, Manassas, VA, USA). Generation of BT474TR and BT474T cells was published elsewhere [3, 13], while 293T cells were provided by A. Stadnyk, Dalhousie University. Lack of mycoplasma contamination in all cells was established as published [14]. BT-474, BT474TR, BT474T, AU-565, MDA-MB-468 and HCC-1806 cells were cultured as per the supplier’s recommendations. 293T cells were cultured as published [14]. To detach cells from the ECM, they were plated above a layer of 1% sea plaque agarose polymerized in respective culture medium not containing additional ingredients.

### Antibodies

Anti-LC3B (cat# 3868S), anti-ATG12 (cat# D88H11), anti-HO-1 (cat# 70081S) and anti-α-tubulin (cat# 3873) were from Cell Signalling Technology. Anti-β-actin (cat # A5316) was from Sigma-Aldrich.

### Cell survival

To count the cells, 30,000 cells were plated in 1 ml of respective medium in a 24-well plate in 2D or 3D culture and treated with dimethylsulfoxide (DMSO) or respective drugs. Cells were further harvested by being incubated at 37 ^0^C in the presence of 50 μl of 0.25% trypsin for 5 min. 50 μl of respective medium was then added to the cells and the cells were counted using a hemocytometer under the light microscope. To measure cell permeability to 7-Amino Actinomycin D (7-AAD) cells were analyzed by use of PE Annexin V Apoptosis Detection Kit I, BD Pharmingen (San Diego, CA, USA) according to manufacturer’s instructions but Annexin V was not used. In brief, the cells were harvested, washed with phosphate buffered saline (PBS) and subjected to centrifugation at 300 × *g* for 5 minutes. This procedure was repeated twice. The cells were further resuspended in 100 μl of the Flow Cytometry Staining Buffer, 5 μl of 7-AAD staining solution was added to each sample, the samples were incubated for 15 min at room temperature in the dark and analyzed by flow cytometry using BD FACS Celesta instrument.

### Detection of LC3 puncta

0.5 × 10^6^ cells were incubated with 1μg of GFP-LC3-encoding expression vector (provided by T. Yoshimori, [15]) and Lipofectamine 3000 (Invitrogen, Carlsbad, CA, USA) according to the manufacturer’s instructions for 48 h. The cells were harvested by trypsinization and seeded at a density of 200,000 cells/well on 18 mm cover glasses placed in a 12-well plate in the case of the 2D culture or in a 12-well plate coated with 1% Sea Plaque agarose in the case of the 3D culture. Cells were further washed with PBS, fixed with methanol for 10 min at room temperature, washed, mounted on the coverslips using fluoromount aqueous mounting medium (Sigma-Aldrich) and imaged using Leica SP8 confocal imager.

### RNA interference

Small interfering (si)RNAs (Horizon Discovery, Lafayette, CO, USA) were utilized as described using Lipofectamin 3000 [16]. HO-1 small hairpin (sh)RNA-encoding lentiviral vectors were from Sigma-Aldrich (St. Louis, MO, USA). HO-1 was knocked down as described [3].

### Orthotopic tumor implantation

Female 6-week-old Nu/Nu Nude mice (Charles River Canada, Saint-Constant, QC) were allowed to acclimatize for 2 weeks. 8 × 10^6^ cells were harvested by trypsin treatment, washed thrice in ice-cold PBS, resuspended in a 100 mL of 1:1 mixture of PBS and Matrigel and injected into the inguinal mammary fat pad. When tumor volumes reached the average of 50 mm^3^, mice were injected intraperitoneally (IP) with either DMSO or 30 mg/kg erastin every other day for 21 days. Tumor volumes were measured as published [17].

### Statistical analysis

Statistical analysis of the data in Supplementary Figs. 1B, E, G and 3B, C, E, G–I was performed by the two-sided chi-square test for goodness-of-fit and statistical analysis of all other data, by the two-sided Student’s *t*-test.

Western blotting [18] and quantitative PCR (qPCR) [3] were performed as published. Western blot quantification was performed by Odyssey or ImageJ software. Original western blots are presented as supplementary material.

Sequences of siRNAs and shRNAs used in the study are shown in Supplementary Table 1.

## Results

### Ferroptosis-promoting treatments block BC cell tumorigenicity in vivo

**Fig. 1:**
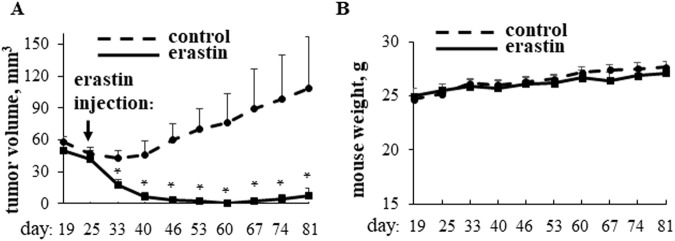
Erastin blocks breast cancer cell tumorigenicity in mice. Immunodeficient Nu/Nu nude mice were injected in the mammary fat pad with BT-474T cells on day 0. When tumor volumes reached the average of 50 mm^3^, mice were injected intraperitoneally with either DMSO (control) or 30 mg/kg erastin every other day for 21 days. 8 mice were used per group (one mouse from the control group had to be sacrificed on day 74 due to vaginitis). Changes in tumor volumes (**A**) and mouse weight (**B**) plus SE are shown. The time of the first injection is indicated by an arrow. *p < 0.05.

We first tested whether a ferroptosis-inducing agent erastin blocks BC cell tumorigenicity in mice. We chose erastin since ferroptosis was discovered based on the use of this drug [19], i.e., its ability to promote ferroptosis is well established. ErbB2/Her2 oncoprotein is often overproduced by BC cells [20] and identification of novel approaches for ErbB2-positive BC treatment is one of the directions of our research [3, 14]. Hence, we examined the effect of erastin on the tumorigenicity of BC cells BT-474T, a variant of ErbB2/Her2-positive human BC cells BT-474 selected for increased tumorigenic capacity in immunodeficient mice [3]. We found that erastin strongly reduced tumorigenicity of these cells (Fig. 1A). Of note, erastin did not seem to be toxic to the mice as they did not lose weight (Fig. 1B) and did not display any gross physical abnormalities. The latter data are consistent with observations made by others that erastin, when injected via the same route at the same dose and frequency for the same time period as in our study is not toxic to the major organs of immunodeficient mice, such as lungs, liver, spleen and kidneys as determined by the examination of the sections of these organs after paraffin embedding and hematoxylin and eosin staining [21]. Similarly, other studies did not detect erastin toxicity to the indicated mouse organs [22, 23].

### 3D growth sensitizes BC cells to ferroptosis-inducing treatments

**Fig. 2:**
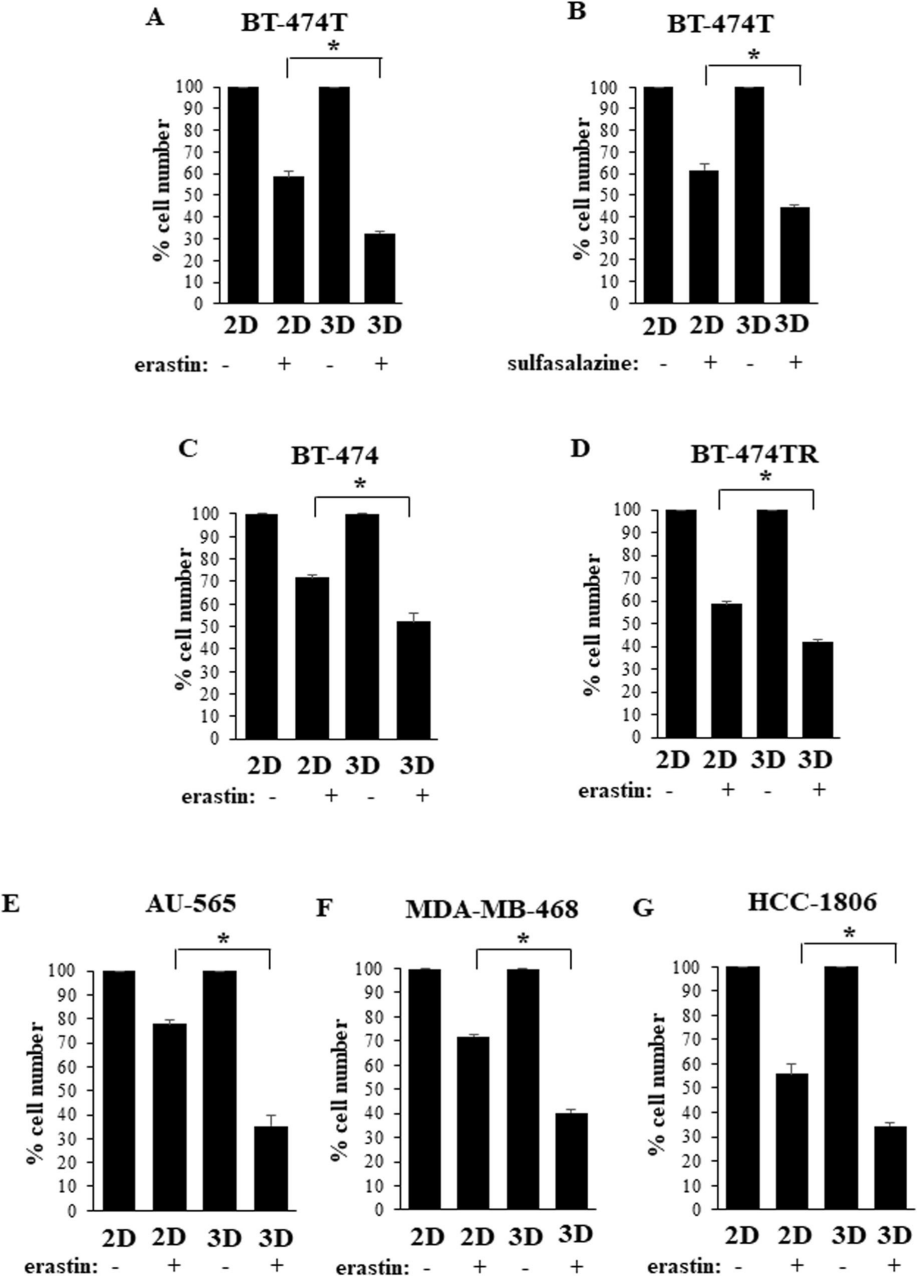
Ferroptosis-inducing drugs block growth breast cancer cells in 3D culture more effectively than in 2D culture. Indicated cell lines were placed in 2D (2D) culture or 3D (3D) culture for 120 h in the presence of DMSO (−) or 10μM erastin (+) (**A**, **C**–**G**) or 200 μM sulfasalazine (**B**) and counted. The data in (**A**–**G**) are the average of three independent experiments plus SD. *p < 0.05.

**Fig. 3:**
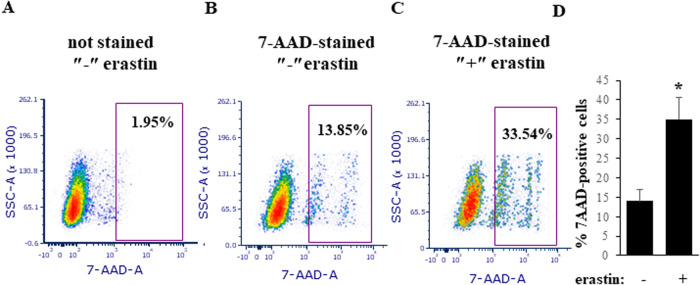
Erastin increases breast cancer cell plasma membrane permeability in 3D culture. BT-474TR cells were placed in 3D culture for 120 h in the presence of DMSO (−) or 10μM erastin (+), and percentage of 7-AAD-positive cells was determined by flow cytometry. Representative flow cytometry plots are shown for erastin-untreated unstained cells (**A**), erastin-untreated 7-AAD-stained cells (**B**), erastin-treated 7-AAD-stained cells (**C**), and the data representing the average of three independent experiments plus SD are shown in (**D**). *p < 0.05.

Ferroptosis mechanisms are understood in the context of BC only in part [24]. In an effort to understand them better, we tested whether erastin inhibits growth of BT-474T cells in two-dimensional (2D) culture when the cells are attached to the tissue culture dish. We treated the cells with 10μM erastin, a concentration well known to effectively inhibit system X_C_^−^ in other cell types and potently kill them [19, 25, 26] but found that erastin reduced such growth relatively modestly (Fig. 2A). Since BCs grow in vivo as three-dimensional 3D masses [2], we tested the effect of erastin on the cells in 3D culture, where the cells grow above a layer of Sea Plaque agarose and form multicellular spheroid-like aggregates [3]. We found that erastin-induced inhibition of 3D cell growth was significantly more pronounced [19] (Fig. 2A). This effect was not unique to erastin since sulfasalazine, another drug that causes ferroptosis by blocking system X_C_^−^ [6], reduced growth of BT-474T cells in 3D culture more efficiently than in 2D culture (Fig. 2B). We observed similar effects in the case of erastin-treated parental BT-474 cells (Fig. 2C). Of note, ErbB2/Her2-positive BC is typically treated with the anti-ErbB2 antibody trastuzumab [27] and often develops trastuzumab resistance [28]. Patients with trastuzumab-resistant tumors invariably die of BC [28]. We found that erastin blocks 3D growth of BT-474TR cells, a trastuzumab-resistant variant of BT-474 cells that we generated [13], more effectively than their 2D growth (Fig. 2D). Hence, ferroptosis induction represents a potential strategy for blocking trastuzumab-resistant BC. The observed effects were not unique to BT-474 cells and their variants since erastin reduced growth of AU-565 cells, another ErbB2-positive human BC cell line [14], in 3D culture more effectively than in 2D culture (Fig. 2E). Moreover, these effects were not unique to ErbB2-positive BC since erastin had a similar impact on MDA-MB-468 and HCC-1806 cells (Fig. 2F, G) derived from the triple-negative BCs lacking ErbB2, estrogen and progesterone receptors [29]. Since ferroptosis represents a type of programmed necrosis [4], we verified by flow cytometry that erastin treatment of BT-474TR cells in 3D culture increases their permeability to a dye 7-AAD, an established necrosis sign [30] (Fig. 3). In summary, BC cells are significantly more sensitive to ferroptosis-inducing drugs in 3D culture than in 2D culture. Even though it is possible that BC sensitivity to these agents in vivo is determined by multiple factors, our data are consistent with the scenario that three-dimensionality of BC growth is at least one of these factors.

### Ferroptosis-promoting treatments trigger autophagy of BC cells in 2D culture more effectively than in 3D culture

**Fig. 4:**
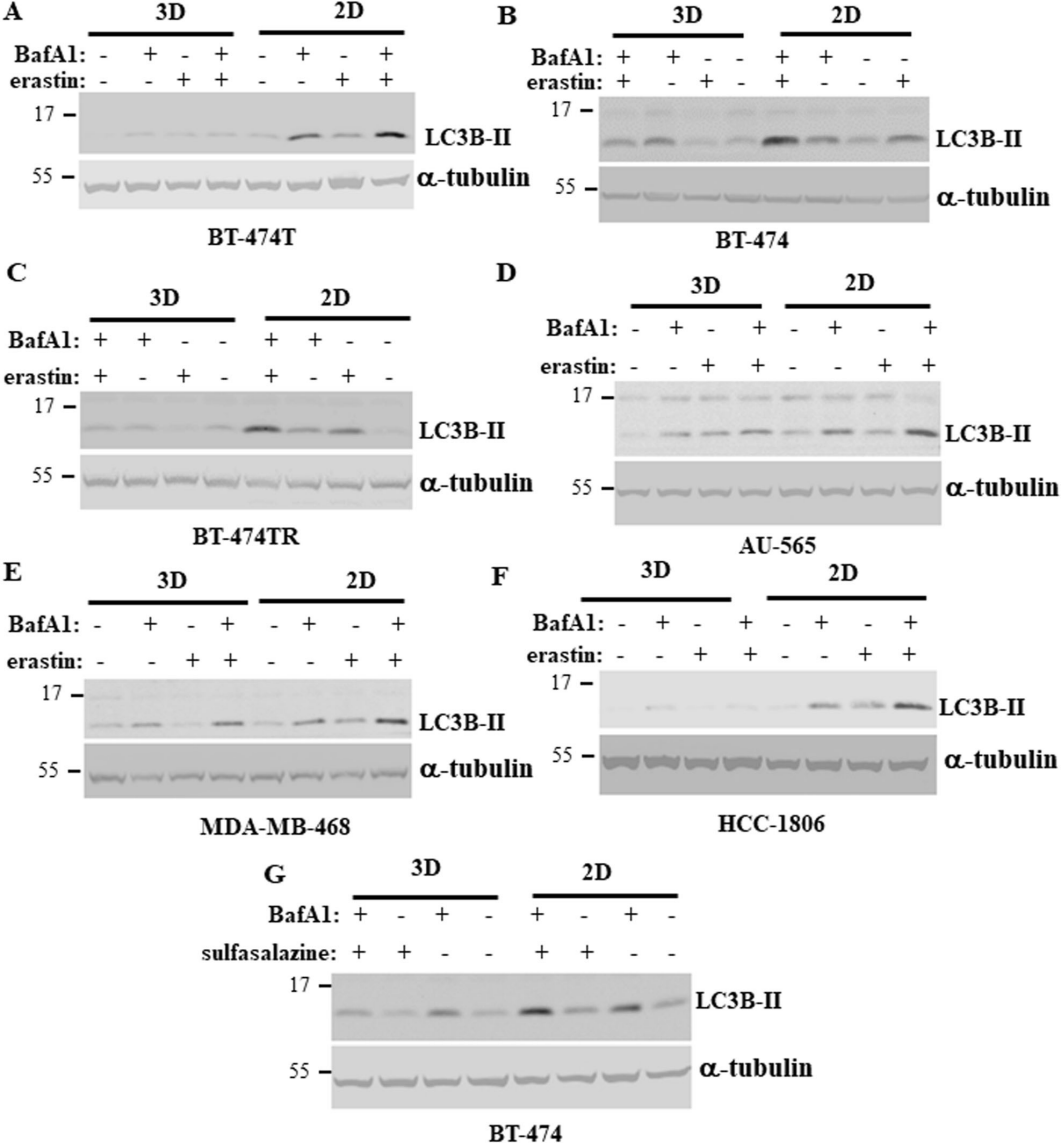
Ferroptosis-inducing drugs cause LC3 lipidation in breast cancer cells in 2D culture more effectively than in 3D culture. Indicated cell lines were placed in 2D (2D) or 3D (3D) culture for 24 h in the presence of DMSO (−) or 10μM erastin (+) (**A**–**F**) or 200 μM sulfasalazine (**G**), 50 nM bafilomycin A1 was added (+) or not (−) to the cells for the last 5 h of the experiment and the cells were assayed for LC3B levels by western blotting. α-tubulin was used as a loading control.

**Fig. 5:**
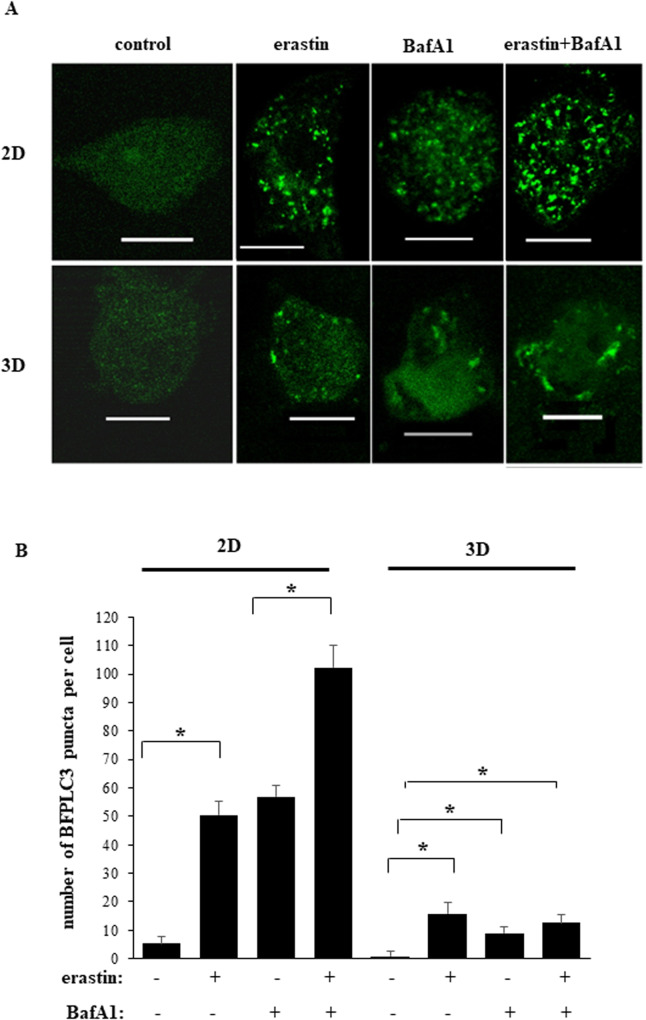
Erastin promotes LC3 puncta formation in breast cancer cells in 2D culture more effectively than in 3D culture. BT-474TR cells were transfected with the GFP-LC3 expression vector, placed in 2D (2D) or 3D (3D) culture for 24 h in the presence of DMSO (control) or 10 μM erastin (+), 50 nM bafilomycin A1 was added (+) or not (−) to the cells for the last 5 h of the experiment and green puncta per cell were counted. **A** Representative fluorescence microscopy images are shown. Bar - 10 μm. **B** Quantification of the number of green puncta per cell for the cells treated as in (A) with DMSO (−), erastin (+) and/or bafilomycin A1 (+). The numbers represent the average of the number of puncta per cell observed for 15 cells plus the SE. This experiment was repeated twice with similar results. *p < 0.05.

We further searched for signals that mediate the differential effect of ferroptosis-inducing drugs in 2D and 3D cultures. Autophagy, a process of degradation of cellular content [7], can block or promote ferroptosis, depending on the context [8, 9, 12]. Hence, we tested whether the level of erastin-induced BC cell autophagy differs between 2D and 3D cultures. Lipidation of autophagy-promoting protein LC3B mediates autophagy, and formation of the lipidated LC3B form called LC3B-II is a well-known autophagy indicator [7]. Noteworthily, LC3B-II is ultimately degraded during autophagy further to autophagosome-to-lysosome fusion. Therefore, while LC3B-II upregulation can be a sign of increased autophagy, it can also signify autophagy inhibition caused by reduced autophagosome-to-lysosome fusion [31]. To distinguish between these scenarios, we tested the effect of erastin on LC3B-II in the presence of bafilomycin A1, a lysosomal inhibitor that prevents the fusion and subsequent LC3B-II degradation [31]. We observed that bafilomycin A1 noticeably upregulated LC3B-II in all BC cell lines tested by us in 2D culture while erastin treatment upregulated LC3B-II in all bafilomycin A1-treated cells even further (Fig. 4A–F). These data indicate that erastin indeed promotes autophagy of BC cells in 2D culture. Remarkably, erastin-dependent LC3B-II upregulation was significantly reduced in 3D culture compared to what we observed in 2D culture in all cell lines tested by us (Fig. 4A–F; see Supplementary Fig. 1A–F for western blot quantification). These findings were not unique to erastin treatment since we observed a similar effect in the case of treatment of BT-474 cells with sulfasalazine, another ferroptosis inducer [4] (Fig. 4G; see Supplementary Fig. 1G for western blot quantification). To verify our findings by a complementary approach, we measured the ability of green-fluorescent protein (GFP)-tagged LC3B protein (GFPLC3) to promote puncta formation in BC cells before and after erastin and/or bafilomycin A1 treatment. Formation of such puncta is an established symptom of autophagosome formation [31]. We observed that bafilomycin A1 treatment of BT-474TR cells transiently transfected with a GFPLC3-encoding expression vector strongly increased the number of the puncta per cell in 2D culture (Fig. 5A, B). Moreover, erastin treatment increased formation of the GFPLC3 puncta in bafilomycin A1-treated cells further (Fig. 5A, B). We also noticed that the ability of erastin, to promote GFPLC3 puncta formation in bafilomycin A1-treated cells was dramatically reduced in 3D culture (Fig. 5A, B). Thus, ferroptosis-promoting drugs trigger autophagy of BC cells when they grow in a 2D manner. When the cells grow in a 3D manner, the ability of ferroptosis-inducing drugs to trigger autophagy of such cells is strongly reduced.

### Erastin-induced autophagy protects BC cells growing in a 2D manner from erastin treatment

**Fig. 6:**
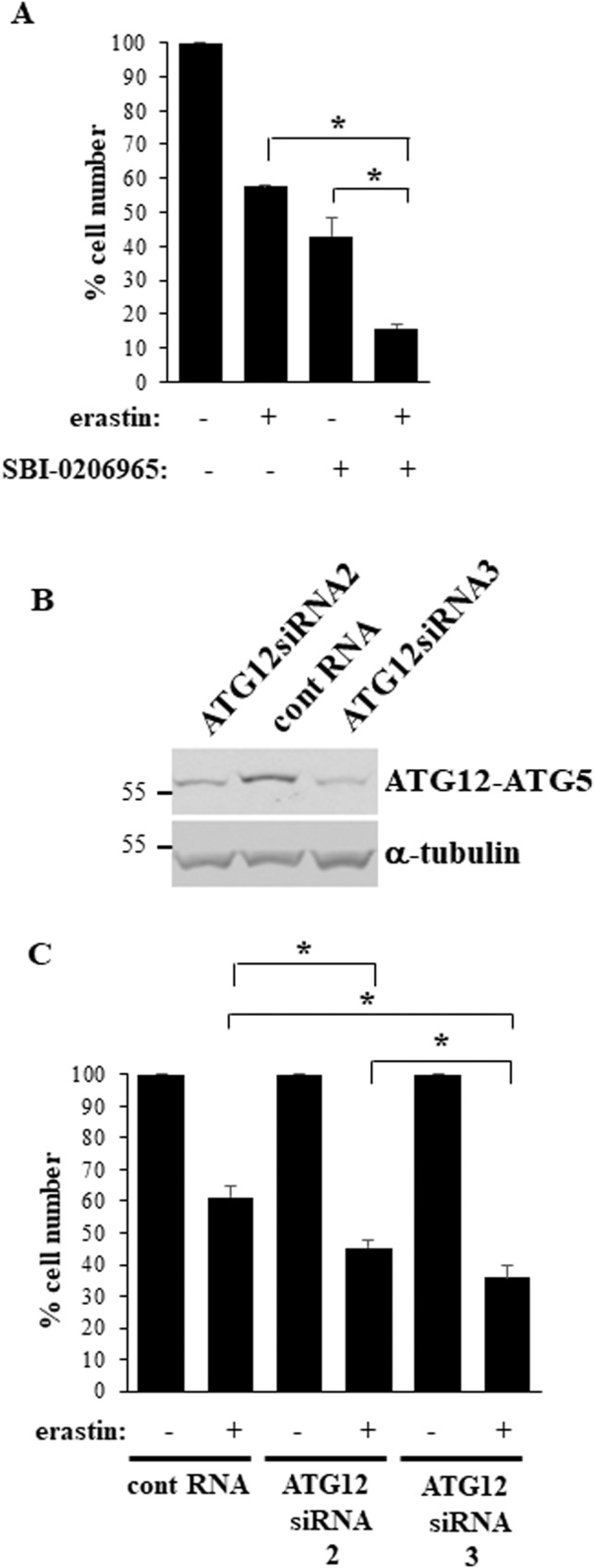
Autophagy inhibition sensitizes breast cancer cells growing in a 2D manner to erastin treatment. **A** BT-474TR cells were placed in 2D culture in the presence of DMSO (−), or 10μM erastin (+) and/or or 2.5μM SBI-0206965 (+) for 120 h and counted. **B** BT-474TR cells were transfected with a control RNA (cont RNA) or ATG12-specific siRNA (ATG12siRNA) 2 or 3 and assayed for the expression of the ATG12-ATG5 conjugate by western blotting. α-tubulin was used as a loading control. **C** BT-474TR cells were transfected as in (B), placed in 2D culture in the presence of DMSO (−) or 10μM erastin (+) for 120 h and counted. The data in (**A**, **C**) represent the average of three independent experiments plus SD. *p < 0.05.

To establish the role of autophagy in survival of BC cells treated with ferroptosis-inducing drugs in 2D culture we treated BT-474TR cells cultured in this manner with erastin in the absence and in the presence of SBI-0206965, a small molecule inhibitor of a protein kinase ULK1, a major component of the cellular autophagy-promoting machinery [32]. We found that SBI-0206965 strongly sensitized BC cells to erastin treatment in 2D culture (Fig. 6A). We further knocked down ATG12, another key component of the autophagy-promoting machinery [7], in BT-474TR cells using two separate ATG12-specific small interfering (si)RNAs (Fig. 6B). The majority of cellular ATG12 is typically covalently bound to ATG5 [33], another autophagy mediator, and the ATG12-ATG5 conjugate is an autophagy driver [31]. We found that both siRNAs substantially downregulated the conjugate in the cells. Noteworthily, the effect of ATG12siRNA3 was more pronounced than that of ATG12siRNA2 (Fig. 6B; see Supplementary Fig. 2 for western blot quantification). Furthermore, both siRNAs significantly sensitized the cells to erastin treatment in 2D culture (Fig. 6C), and the effect of ATG12siRNA3 was more noticeable than that of ATG12siRNA2 (Fig. 6A, B). Thus, erastin-induced autophagy protects BC cells growing in a 2D manner from erastin-dependent death.

### Erastin upregulates mRNAs encoding multiple autophagy inducers in BC cells growing in a 2D but not in a 3D manner

**Fig. 7:**
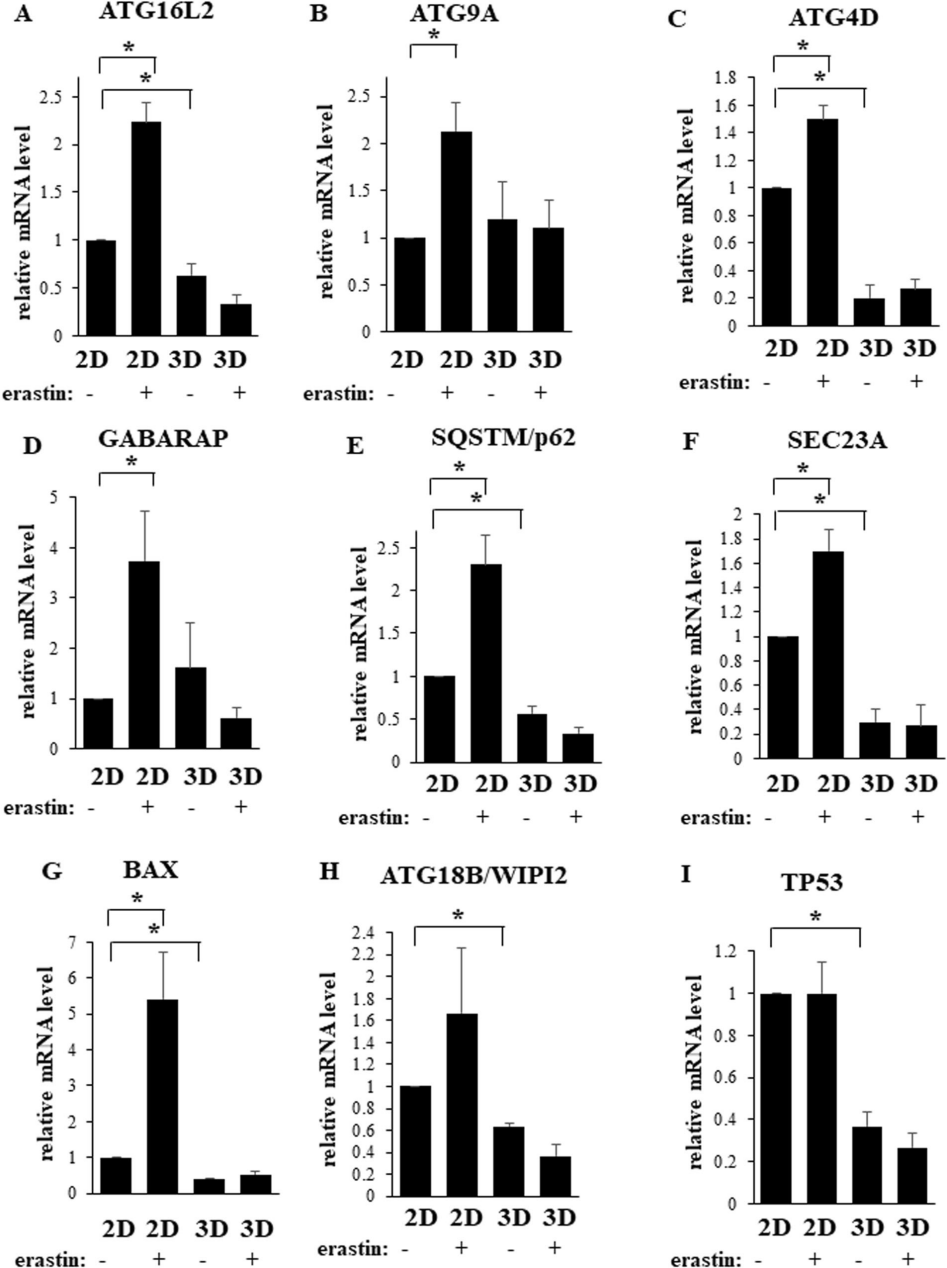
Erastin upregulates mRNAs encoding multiple autophagy inducers in breast cancer cells growing in a 2D but not in a 3D manner. BT-474 cells were placed in 2D (2D) or 3D (3D) culture for 24 h in the presence of DMSO (−) or 10μM erastin (+) and assayed for the expression of the indicated mRNAs by qPCR. The resulting mRNA levels were normalized by those of GAPDH-encoding mRNA (also determined by qPCR). The resulting number observed in the case of the untreated cells in 2D culture was designated as 1.0. Results represent the average of three independent experiments plus the SD. *p-value < 0.05.

To gain insights into the mechanisms by which 3D growth of BC cells prevents ferroptosis-inducing drugs from triggering autophagy we used a commercially available set of primers for 88 mRNAs encoding direct and indirect autophagy regulators and detected these mRNAs by quantitative PCR in BT474 cells treated or not with erastin in 2D and 3D culture. We found that erastin significantly upregulated mRNAs encoding autophagy stimulators ATG16L2, ATG9A, ATG4D, GABARAP, SQSTM/p62, SEC23A and BAX in the cells growing in 2D but not in 3D culture (Fig. 7A–G). ATG16L2, ATG9A, ATG4D, GABARAP and SQSTM/p62 are the components of the cellular autophagy-promoting machinery [31]. Sec23A is constituent of coat protein complex II that promotes autophagy via cellular transport of a multi-functional protein S100A8 [34], while an apoptosis inducer BAX triggers autophagy via poorly understood mechanisms [35]. We further noticed that the cells growing in 2D culture displayed substantially higher levels of mRNAs encoding ATG16L2, ATG4D, SQSTM/p62, SEC23A, BAX, ATG18B/WIPI2 and TP53 than the cells growing in 3D culture (Fig. 7A, C, E–I). ATG18B/WIPI2 is the component of the cellular autophagy-promoting machinery [31] while transcription activator TP53 (which is functional in BT474 cells [36]) promotes autophagy via multiple mechanisms [37]. Collectively, our data are consistent with the scenario that 3D growth prevents ferroptosis-inducing drugs from triggering multiple autophagy-promoting mechanisms in BC cells and downregulates multiple autophagy mediators in such cells thereby reducing the capacity of the cells for activating autophagy in response to these agents.

### HO-1 mediates ferroptosis of BC cells growing in a 3D manner

**Fig. 8:**
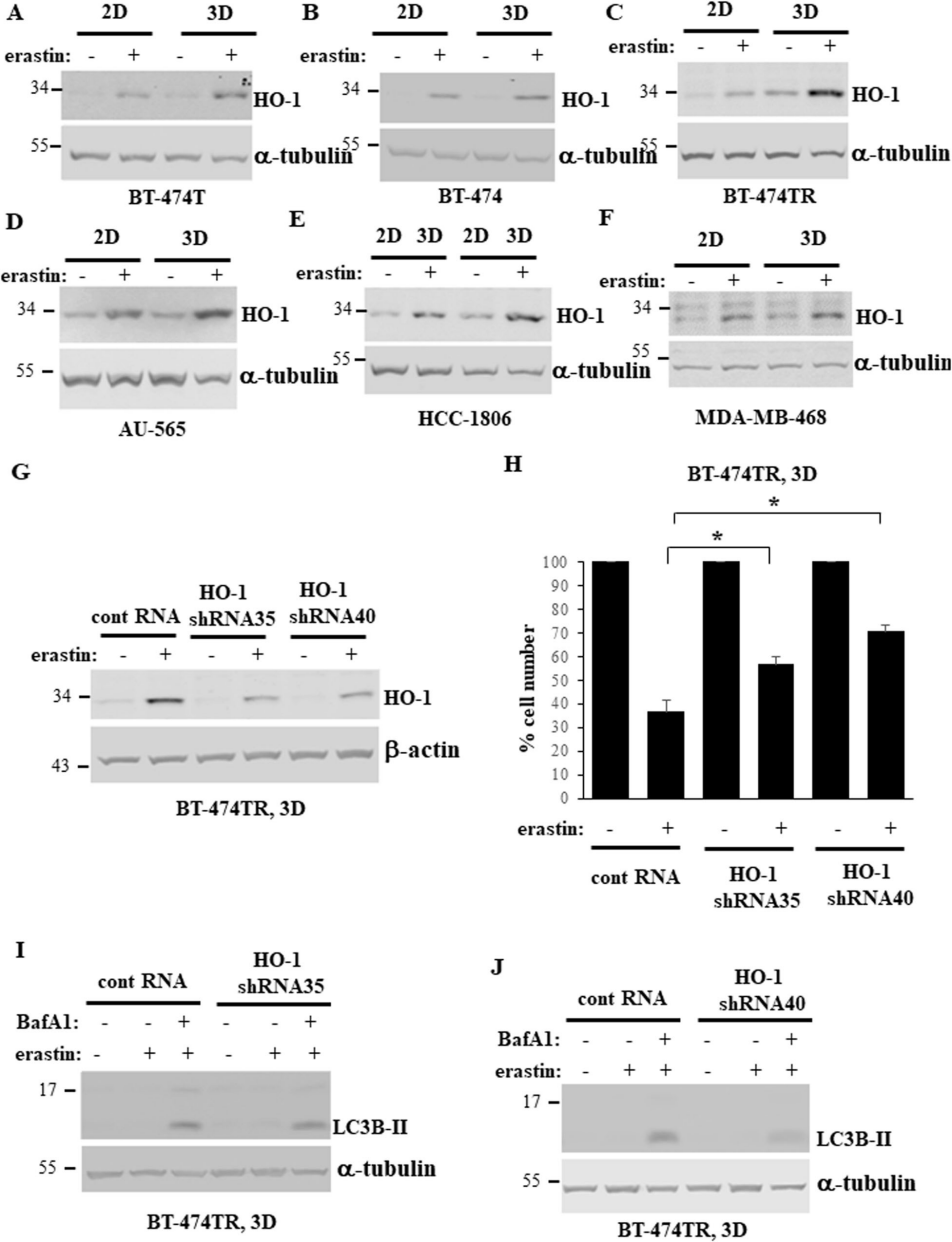
HO-1 mediates ferroptosis of breast cancer cells growing in a 3D manner. **A**–**F** Indicated cell lines were placed in 2D (2D) or 3D (3D) culture for 24 h in the presence of DMSO (−) or 10μM erastin (+) and assayed for HO-1 expression by western blotting. α-tubulin was used as a loading control. **G** BT474-TR cells were infected with a control lentivirus or that encoding HO-1-specific shRNA (HO-1shRNA) 35 or 40, placed in 3D (3D) culture for 24 h in the presence of DMSO (−) or 10μM erastin (+) and assayed for HO-1 expression by western blotting. β-actin was used as a loading control. **H** BT474-TR cells processed as in (**G**) were placed in 3D culture in the presence of DMSO (−) or 10μM erastin (+) for 120 h and counted. The data represent the average of the triplicates plus SD. *p < 0.05. This experiment was repeated twice with similar results. **I**, **J** BT474-TR cells processed as in (**G**) were placed in 3D culture for 24 h in the presence of DMSO (−) or 10 μM erastin (+), 50 nM bafilomycin A1 was added (+) or not (−) to the cells for the last 5 h of the experiment, and the cells were assayed for LC3B levels by western blotting. α-tubulin was used as a loading control.

According to our data, ferroptosis-inducing drugs represent promising tools for blocking BC 3D growth. Molecular pathways regulating ferroptosis are understood only in part [38] and the mechanisms by which ferroptosis-inducing agents block 3D growth of BC cells are not known. Hence, we decided to investigate them. Heme oxygenase-1 (HO-1) is an enzyme that degrades the Fe^2+^-containing compound heme to generate biliverdin-IXα and free Fe^2+^ [39]. Ferroptosis-inducing drugs, such as erastin, upregulate HO-1 in cells by increasing HO-1 mRNA levels via a complex set of signals involving downregulation of the transcriptional repressor BACH1 and upregulation of the transcription factor NRF2 [40, 41]. In some circumstances, HO-1 protects cells from ferroptosis-associated oxidative stress, e.g., due to the anti-oxidant properties of some of the heme degradation products [39]. However, in other contexts, Fe^2+^ released after HO-1-dependent heme degradation amplifies lipid ROS-induced death signals [42, 43]. Factors determining when HO-1 blocks and when it promotes ferroptosis are not well understood. Whether HO-1 regulates ferroptosis of BC cells growing in a 3D manner is unknown. We found that erastin upregulates HO-1 in all BC cell lines tested by us both in 2D and 3D culture (Fig. 8A–F; see Supplementary Fig. 3A–F for western blot quantification). Moreover, HO-1 knockdown by two different shRNAs significantly protected BT-474TR cells in 3D culture from erastin-induced death (Fig. 8G, H; see Supplementary Fig. 3G for western blot quantification). These data are consistent with the scenario that HO-1 upregulation contributes to ferroptosis of BC cells growing in a 3D manner. Noteworthily, HO-1 knockdown did not affect LC3B-II levels in the cells treated with bafilomycin A1 and erastin in 3D culture (Fig. 8I, J; see Supplementary Fig. 3H, I for western blot quantification) indicating that HO-1 promotes their death without blocking autophagy (the levels of which are already relatively low in these cells in 3D culture, see Figs. 4 and 5).

In summary, we found that ferroptosis-inducing drugs suppress BC cell 2D growth relatively ineffectively because these agents trigger pro-survival autophagy. In contrast, the ability of these agents to trigger autophagy in the cells growing in a 3D manner is substantially reduced. Ferroptosis inducers kill such cells significantly more effectively, and this death is likely mediated by HO-1. Furthermore, erastin, one of these agents, strongly suppresses BC cell tumorigenicity. Thus, ferroptosis-promoting drugs are promising tools for blocking 3D BC growth.

## Discussion

We show here that ferroptosis inducer erastin, strongly inhibits BC cell tumorigenicity. Moreover, BC cells grow in vivo as 3D masses [2], and we found that ferroptosis-promoting drugs block 3D growth of BC cells more effectively than 2D growth.

The impact of anti-cancer drugs on tumor cells is often studied in 2D culture, a model that does not reliably predict the efficacy of these drugs in vivo [44, 45]. In contrast, the 3D culture models are thought to mimic tumor architecture and signalling events acting in tumor cells more adequately [45, 46]. Indeed, our data obtained in 3D culture correlate with the effect of ferroptosis-inducing drugs in vivo significantly better than those obtained in 2D culture.

We found that ferroptosis inducing drugs trigger pro-survival autophagy in the cells in 2D but not in 3D culture. In some models, e.g., fibroblasts, autophagy was proposed to drive ferroptosis [12]. In other models, e.g., those based on lung cancer, glioblastoma, hepatocellular carcinoma cells and neurons, autophagy protects the cells from ferroptosis-inducing drugs [8–11]. Our data clearly indicate that in the context of BC, autophagy triggered by ferroptosis inducers in the cells growing in a 2D manner protects them from death. Conceivably, ferroptosis-inducing drugs trigger autophagy in such cells as a feedback mechanism that helps them to protect themselves from ferroptosis-associated oxidative stress, e.g., by eliminating molecules and/or organelles damaged during ferroptosis.

We found for the first time that ferroptosis inducers strongly promote autophagy of BC cells growing in a 2D manner but fail to do so in the cells growing in a 3D manner. These data are consistent with observations made by others that in other contexts, a triple-negative BC cell line [47], several mesothelioma cell lines [48] and mesenchymal stem cells [49] growing in a 3D manner have lower capacity to induce autophagy than the cells growing in a 2D manner. We established that while a ferroptosis inducer erastin noticeably upregulated the mRNAs encoding multiple autophagy-promoting proteins in the cells growing in 2D culture it failed to do so in the cells growing in a 3D manner. These data are consistent with a scenario that ferroptosis inducers trigger autophagy in 2D culture via multiple mechanisms which are inhibited when 3D tumor cell mass is formed.

Unlike the cells in 3D culture, cells growing in a 2D manner are attached to the ECM that they deposit on the culture dish surface [3]. Moreover, unlike the cells in 2D culture, cells growing in a 3D manner form multicellular spheroid-like aggregates where numerous cell-to-cell contacts are formed [45]. Establishing whether loss of the ability of ferroptosis inducers to promote autophagy is caused by cancer cell detachment from the ECM, the formation of the multicellular 3D aggregates or both types of events represents an important direction for our future studies.

Our data support the scenario that HO-1 upregulation caused by ferroptosis-inducing drugs in BC cells growing in a 3D manner mediates their death. These results are consistent with findings made by others that HO-1 promotes BC cell ferroptosis in response to certain drugs [50] and that HO-1-upregulating agents block BC progression in vivo [51].

BCs often develop resistance to therapies, and patients typically die of the resulting metastatic disease [1]. We found that ferroptosis-inducing drug erastin substantially inhibits 3D growth of ErbB2-positive BC cells that developed resistance to ErbB2/Her2-targeted drug trastuzumab. Thus, ferroptosis inducers could potentially improve the existing BC treatments, e.g., by being used together with trastuzumab to delay the onset of BC trastuzumab resistance and/or together with ErbB2-targeted drugs utilized for trastuzumab-resistant BC treatment. In summary, our data indicate that the use of ferroptosis-promoting agents represents a promising strategy for blocking 3D BC growth.

## Supplementary information

Supplementary figures

Supplementary table 1

Reproducibility checklist

Original western blots

## Data Availability

All data reported in this study will be shared by us upon request.
