# Supplementary material for: Three-dimensional growth sensitizes breast cancer cells to treatment with ferroptosis-promoting drugs
Source: Cell Death Dis. 2023 Sep 1;14(9):580. doi: 10.1038/s41419-023-06106-2 (PMC10474142; doi:10.1038/s41419-023-06106-2)
Supplement: Supplementary file 1 — Supplementary figures [file 41419_2023_6106_MOESM1_ESM.docx]

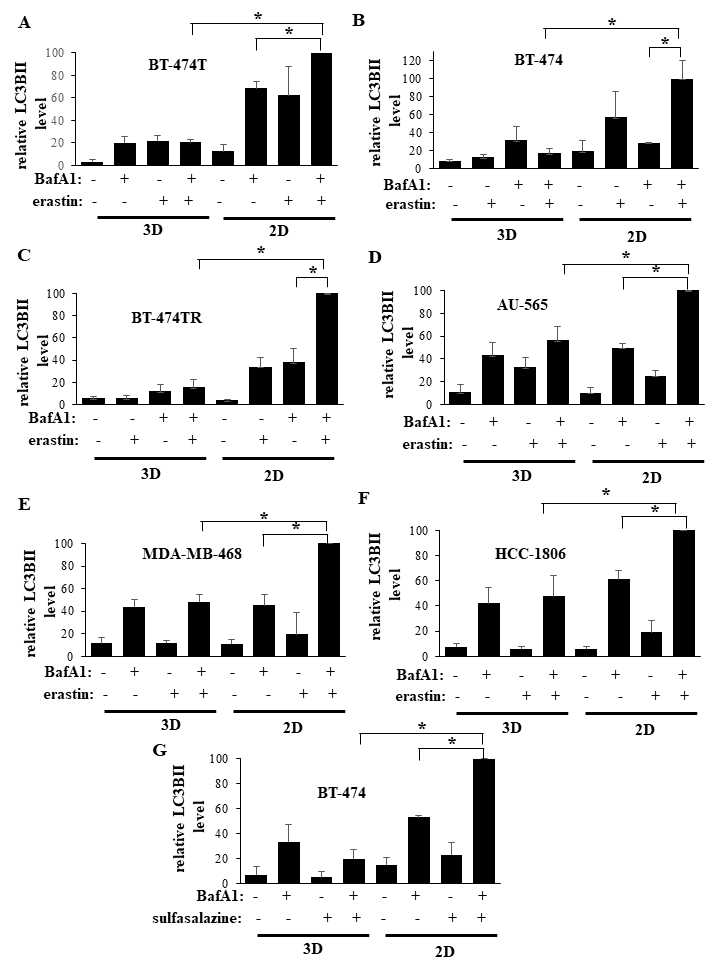


Supplementary fig. 1 Quantification of the western blots shown in Fig. 4. The data in (A-G) represent quantification of the data shown in Fig. 4(A-G), respectively. The data in (A) represent the average of three, the data in (B, E, G) the average of two, and the data in (C, D, F) the average of four independent experiments plus SE. LC3BII protein levels were normalized by those of the respective loading control in the case of each experiment. * - p˂0.05.


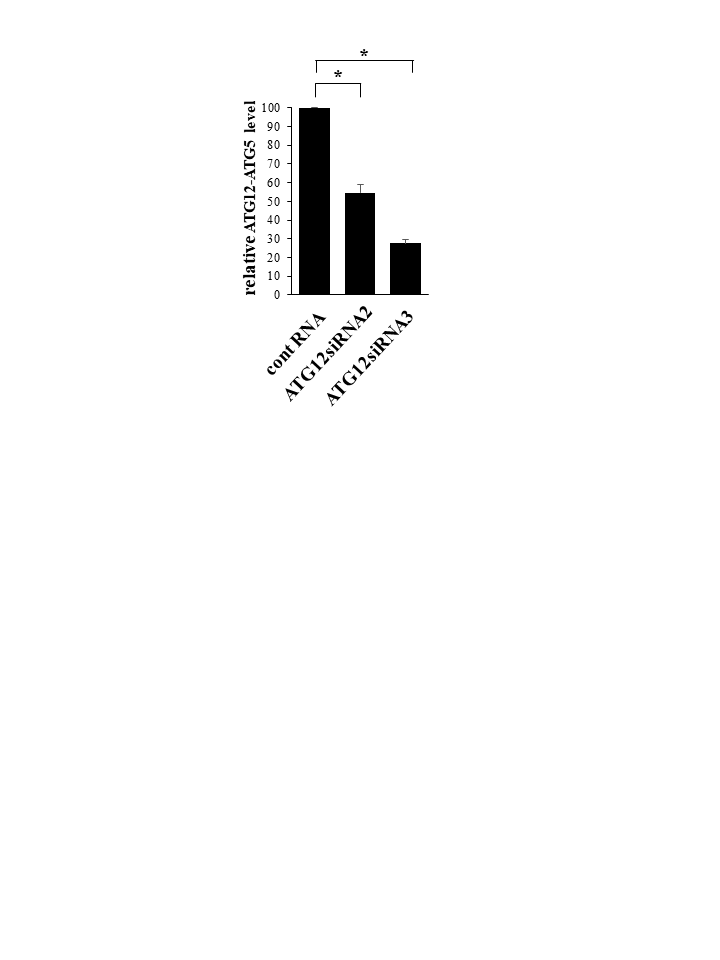


Supplementary fig. 2 Quantification of the western blots shown in Fig. 6B. The data represent the average of three independent experiments plus SE. ATG12 protein levels were normalized by those of the respective loading control in the case of each experiment. * - p˂0.05.


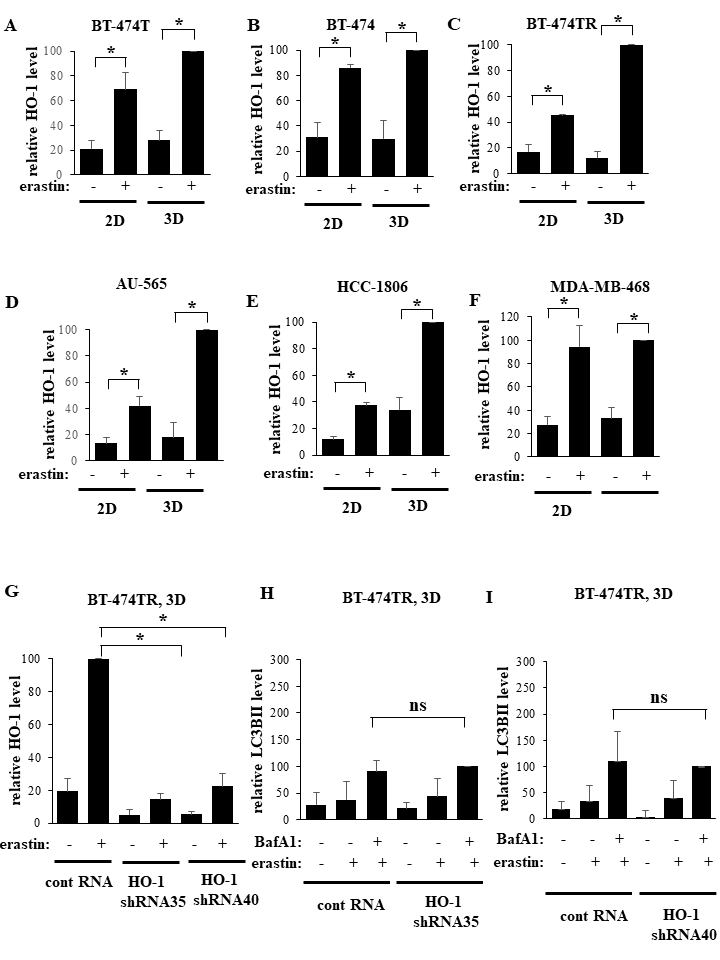


Supplementary fig. 3 Quantification of the western blots shown in Fig. 8. The data in (A-G) represent quantification of the data shown in Fig. 8(A-G), respectively. The data in (H, I) represent quantification of the data shown in Fig. 8(I, J), respectively. The data in (A, F) represent the average of three, the data in (B, C, E, G-I) the average of two, and the data in (D) the average of four independent experiments plus SE. HO-1 and LC3BII protein levels were normalized by those of the respective loading control in the case of each experiment. * - p˂0.05.
