# Supplementary material for: Three-dimensional growth sensitizes breast cancer cells to treatment with ferroptosis-promoting drugs
Source: Cell Death Dis. 2023 Sep 1;14(9):580. doi: 10.1038/s41419-023-06106-2 (PMC10474142; doi:10.1038/s41419-023-06106-2)
Supplement: Supplementary file 2 — Supplementary table 1 [file 41419_2023_6106_MOESM2_ESM.docx]

ATG12 siRNA2 GGGAAGGACUUACGGAUGU (Horizon Discovery, cat # J-010212-08)

ATG12 siRNA3 GCAGUAGAGCGAACACGAA (Horizon Discovery, cat # J-010212-07)

HO-1 shRNA35 ACAGTTGCTGTAGGGCTTTAT (Sigma Aldrich, cat # TRCN0000290435)

HO-1 shRNA40 CATCCAGGCAATGGCCTAAAC (Sigma Aldrich, cat # TRCN0000296640)

**Supplementary table 1** Sequences of siRNAs and shRNAs used in the study.
