# Supplementary material for: Three-dimensional growth sensitizes breast cancer cells to treatment with ferroptosis-promoting drugs
Source: Cell Death Dis. 2023 Sep 1;14(9):580. doi: 10.1038/s41419-023-06106-2 (PMC10474142; doi:10.1038/s41419-023-06106-2)
Supplement: Supplementary file 4 — Original western blots [file 41419_2023_6106_MOESM4_ESM.docx]

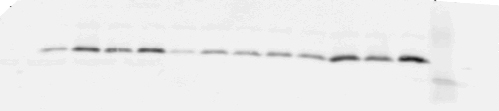


**LC3B-II**


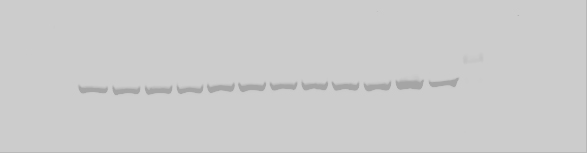


**α-tubulin**


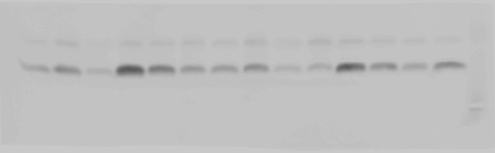
Supplementary fig. 9 Original western blots for the data shown in Fig. 4A. Lanes 5-12 are shown in Fig. 4A.

**LC3B-II**


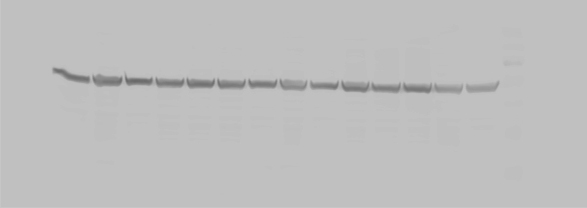


**α-tubulin**


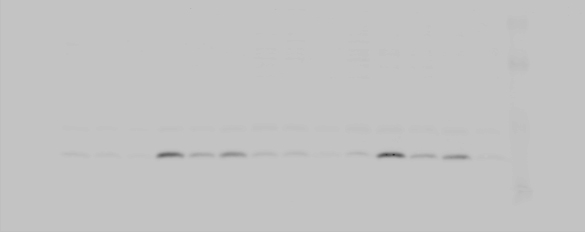
Supplementary fig. 10 Original western blots for the data shown in Fig. 4B. Lanes 7-14 are shown in Fig. 4B.

**LC3B-II**


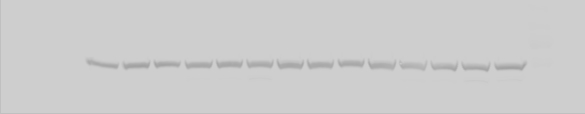


**α-tubulin**

Supplementary fig. 11 Original western blots for the data shown in Fig. 4C. Lanes 7-14 are shown in Fig. 4C


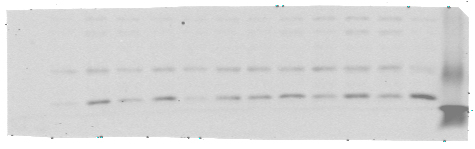


**LC3B-II**


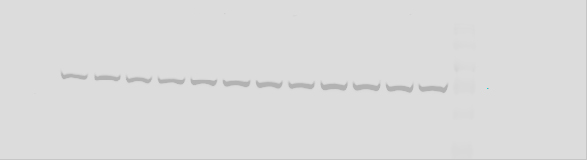


**α-tubulin**

Supplementary fig. 12 Original western blots for the data shown in Fig. 4D. Lanes 5-12 are shown in Fig. 4D.


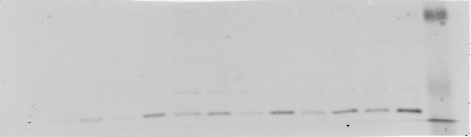


**LC3B-II**


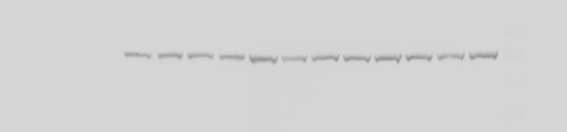


**α-tubulin**

Supplementary fig. 13 Original western blots for the data shown in Fig. 4E. Lanes 5-12 are shown in Fig. 4E.


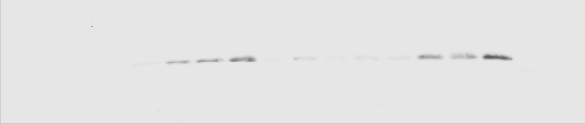


**LC3B-II**


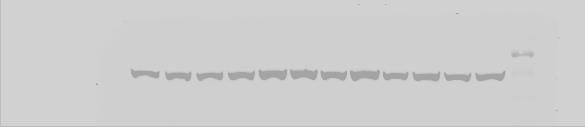


**α-tubulin**

Supplementary fig. 14 Original western blots for the data shown in Fig. 4F. Lanes 5-12 are shown in Fig. 4F.


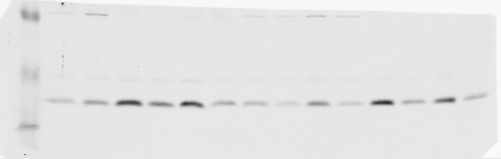
.

**LC3B-II**


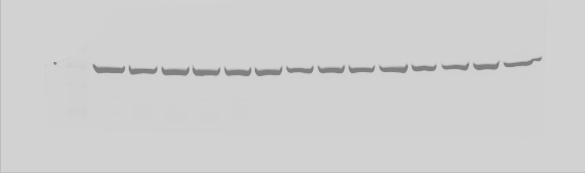


**α-tubulin**

Supplementary fig. 15 Original western blots for the data shown in Fig. 4G. Lanes 6-13 are shown in Fig. 4G


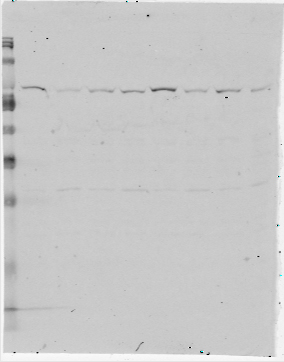


**ATG12-ATG5**


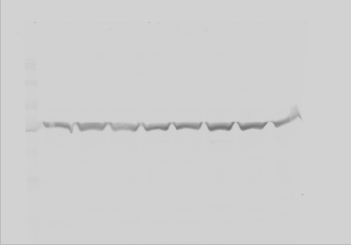


**α-tubulin**

Supplementary fig. 16 Original western blots for the data shown in Fig. 6B. Lanes 4-16 are shown in Fig. 6B.


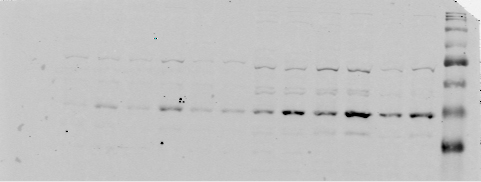


**HO-1**


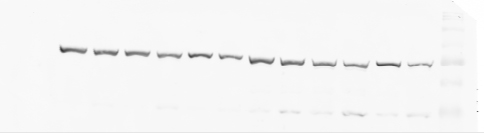

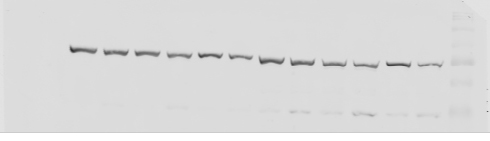


**α-tubulin**

Supplementary fig. 17 Original western blots for the data shown in Fig. 8A. Lanes 1-4 are shown in Fig. 8A.


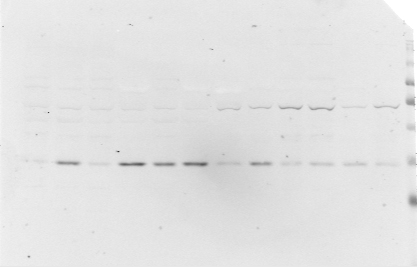


**HO-1**


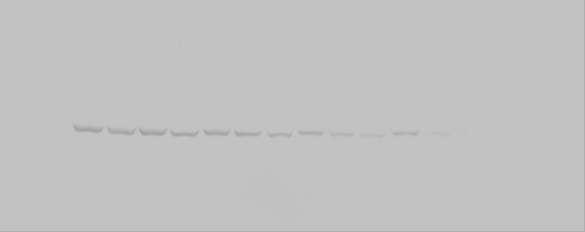


**α-tubulin**

Supplementary fig. 18 Original western blots for the data shown in Fig. 8B. Lanes 1-4 are shown in Fig. 8B.

.


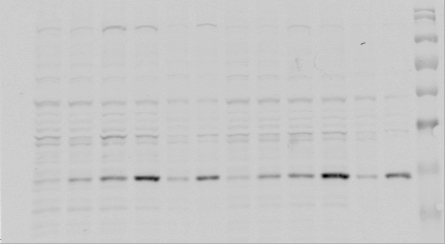


**HO-1**


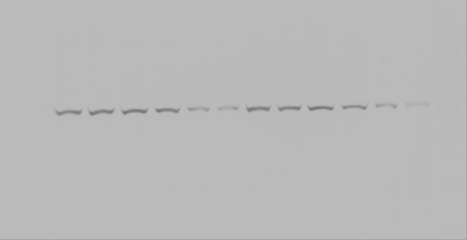


**α-tubulin**

Supplementary fig. 19 Original western blots for the data shown in Fig. 8C. Lanes 7-10 are shown in Fig. 8C.


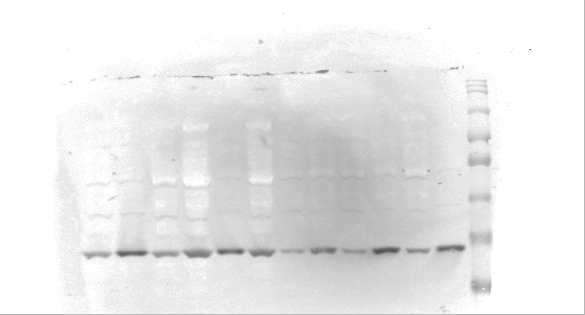


**HO-1**


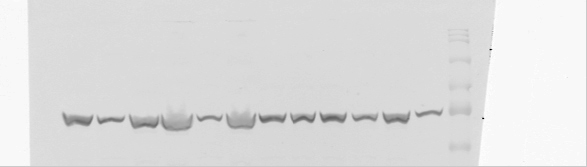


**α-tubulin**

Supplementary fig. 19 Original western blots for the data shown in Fig. 8D. Lanes 7-10 are shown in Fig. 8D.


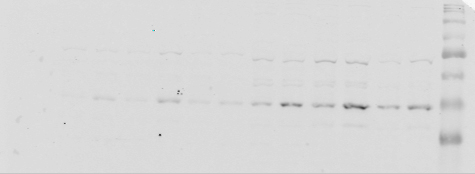


**HO-1**


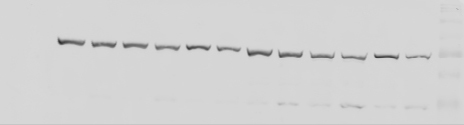


**α-tubulin**

Supplementary fig. 20 Original western blots for the data shown in Fig. 8E. Lanes 7-10 are shown in Fig. 8E


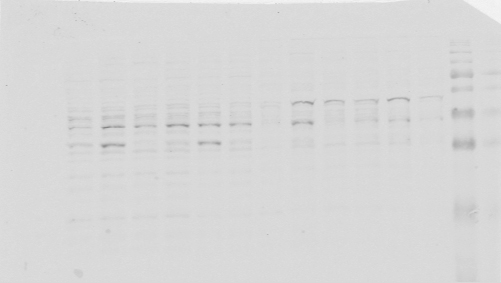


**HO-1**


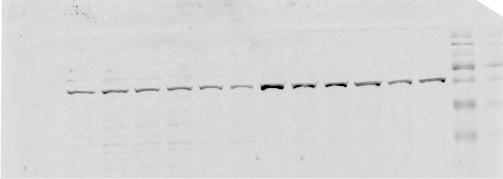


**α-tubulin**

Supplementary fig. 21 Original western blots for the data shown in Fig. 8F. Lanes 1-4 are shown in Fig. 8F.


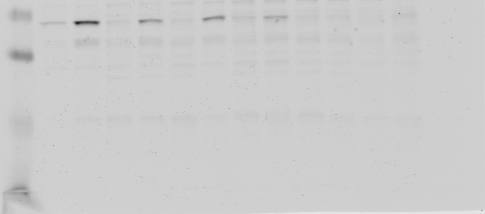


**HO-1**


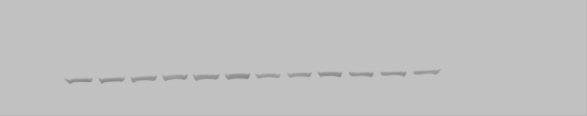


**β-actin**

Supplementary fig. 22 Original western blots for the data shown in Fig. 8G. Lanes 1-6 are shown in Fig. 8G.


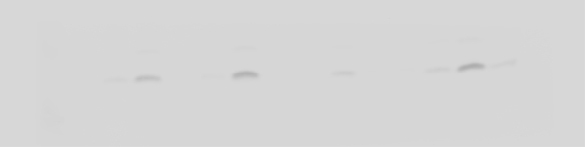


**LC3B-II**


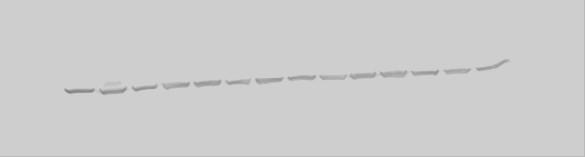


**α-tubulin**

Supplementary fig. 23 Original western blots for the data shown in Fig. 8I. Lanes 7-12 are shown in Fig. 8I.


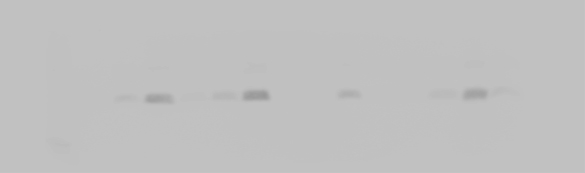


**LC3B-II**


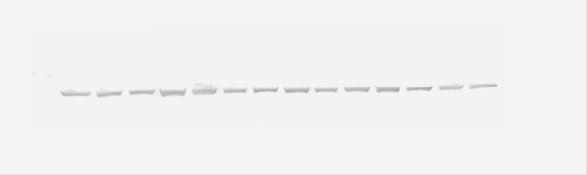


**α-tubulin**

Supplementary fig. 24 Original western blots for the data shown in Fig. 8J. Lanes 7-12 are shown in Fig. 8J.
